# Standardisierte strukturierte Befundberichte gastrointestinaler Tumoren

**DOI:** 10.1007/s00292-021-00986-x

**Published:** 2021-10-05

**Authors:** Ekkehard Hewer, Anna Rump, Rupert Langer

**Affiliations:** 1 Institut universitaire de pathologie, Centre hospitalier universitaire vaudois (CHUV) et Université de Lausanne, Rue du Bugnon 25, 1011 Lausanne, Schweiz; 2 Institut für Pathologie und Molekularpathologie, Kepler Universitätsklinikum und Johannes-Kepler-Universität, Linz, Österreich

**Keywords:** Checkliste, Datengenauigkeit, Qualitätssicherungsmaßnahmen im Gesundheitswesen, Pathologie, Qualitätskontrolle, Checklist, Data accuracy, Healthcare quality assurance, Pathology, Quality control

## Abstract

Synoptische oder strukturierte Berichte in der Medizin, speziell in der Pathologie, sind im Gegensatz zu traditionellen narrativen Berichten gekennzeichnet durch ein listen- bzw. laborwertartiges Format und die Verwendung standardisierter Checklisten. Sie tragen zur Vollständigkeit und Verständlichkeit der Berichte und damit letztlich zu einer verbesserten Patientenversorgung bei. Für die Verwendung in der Pathologie publizieren aktuell 2 größere Institutionen Vorlagen für synoptische Berichte, das College of American Pathologists (CAP) und die International Collaboration for Cancer Reporting (ICCR). Synoptische Berichtsvorlagen sind für alle häufigeren Tumortypen verfügbar. Sie bieten nicht nur eine standardisierte Nomenklatur und Checklisten für vollständige Berichte, sondern unterstützen auch eine einheitliche Anwendung diagnostischer Kriterien. Außerdem beinhalten die Protokolle sowohl von CAP als auch von ICCR umfangreiche und aktuelle Referenzen und sind damit eine wertvolle Quelle von Zusatzinformationen, selbst wenn kein synoptisches Berichtsformat verwendet wird. Nutzen und Herausforderungen bei der Implementation von synoptischen Berichten werden diskutiert, insbesondere im Hinblick auf die deutschsprachige Pathologie.

## Abstract (en)

Synoptic reporting in pathology as opposed to traditional narrative reporting is defined by a laboratory value-like format and use of standardized checklists. It contributes to completeness and comprehensibility of pathology reports and ultimately patient care. As of today, two major institutions publish synoptic reporting templates, the College of American Pathologists (CAP) and the International Collaboration for Cancer Reporting (ICCR). Synoptic protocols are available for all major cancer types and provide not only a standardized terminology and a checklist for completeness of reports, but also facilitate uniform utilization of diagnostic criteria. Additionally, both CAP and ICCR protocols are accompanied by detailed and up-to-date reference lists and thereby represent a valuable source of information even when synoptic reporting is not used. The benefits and challenges of implementation of synoptic reporting are discussed, in particular with regard to reporting in German.

Wer kennt sie nicht: die Anrufe eines klinischen Kollegen, ob man sich nicht bitte noch zu diesem Parameter Stellung nehmen könne, der im Bericht leider fehle, aber doch ungemein wichtig sei. Merkwürdig nur, dass sich keiner seiner Fachkollegen je dafür interessiert und es dazu nur eine einzige Studie von zweifelhafter Qualität gibt – so ist „personalisierte Medizin“ eigentlich nicht gedacht! Nicht nur für derlei Probleme, sondern allgemein für die Frage, was in einen Bericht gehört und wie man es formulieren sollte, bietet das Konzept der synoptischen Berichte interessante Lösungsansätze

Eine Standardisierung von Kommunikation kann einen wichtigen Beitrag zur Sicherheit in der Medizin leisten. Wesentliche Aspekte einer solchen standardisierten Kommunikation sind einerseits die Vollständigkeit der kommunizierten Parameter (z. B. im Rahmen einer Checkliste, die vor Beginn einer Operation im Rahmen eines „team timeout“ durchgegangen wird), andererseits eine eindeutige und unmissverständliche Terminologie. In der Pathologie bietet sich hierfür das Konzept der „synoptischen“ (oder auch „strukturierten“ bzw. „standardisierten“) Berichte an. Anders als z. B. in den Vereinigten Staaten oder den Niederlanden scheinen sie im deutschen Sprachraum bislang wenig verbreitet zu sein, auch wenn systematische Erhebungen hierzu fehlen. Dieser Übersichtsartikel diskutiert das Konzept der standardisierten Berichte, die Datenlage zu deren Nutzen und Limitationen sowie Aspekte der praktischen Umsetzung.

## Was sind synoptische Berichte?

Einem Bonmot zufolge, das ursprünglich auf eine andere Berufsgruppe gemünzt war, sich aber durchaus auf die unsrige übertragen lässt, müsse man sich eine Pathologin oder einen Pathologen vorstellen als eine „Person, die eher die Zahnbürste ihres Kollegen als dessen Terminologie benutzen würde“. In der Tat kennen wir wohl alle Beispiele terminologischer Eigenheiten, die die Verfasserin oder den Verfasser eines Pathologieberichts schnell preisgeben und die nicht selten mit einiger Vehemenz verteidigt werden. Man müsse eben genau so und so sagen, weil man damit etwas Bestimmtes gleich mit einschließe oder weil es eine elegante Möglichkeit sei, sich nicht auf dieses oder jenes festlegen zu müssen. Bei aller Sympathie für eine elaborierte und differenzierte Sprache ist eine uneinheitliche medizinische Terminologie jedoch eine potenzielle Quelle von Missverständnissen und womöglich daraus resultierenden fehlerhaften Entscheidungen.

An diesem Punkt setzt das Konzept der synoptischen Berichte an: Die jeweiligen organ- und /oder tumortypspezifischen Protokolle definieren eine eindeutige Terminologie, nicht nur für jeden der betreffenden diagnostischen oder prognostischen Parameter (z. B. „Dysplasia“), sondern auch für dessen Status („not identified“ oder „present“) sowie für etwaige nähere Spezifizierungen („grade“: „low-grade“ oder „high-grade“; „type“: „squamous“ oder „columnar/barrett“).

Die präziseste Definition synoptischer Berichte stammt vom College of American Pathologists (CAP) und umfasst folgende Aspekte [3]:

- Ein listen- bzw. laborwertähnliches Format für jeden Parameter, bestehend aus der Bezeichnung des Datenelements („required data element“) und der Antwort („response“) darauf.
- Im Allgemeinen steht jedes Datenelement in einer separaten Zeile (bestimmte eng zusammenhängende Elemente wie Tumorlokalisation und Art der Resektion können in einer Zeile zusammengefasst werden).
- Der Bericht muss alle obligatorischen Datenelemente (gemäß dem jeweiligen Protokoll) enthalten.

Hingegen werden keine Vorgaben gemacht zur Reihenfolge der Datenelemente, zu etwaigen zusätzlichen Elementen, zu ergänzenden Abschnitten in narrativem Format (d. h. in fortlaufendem Text) oder zu weiteren Aspekten der Formatierung.

## Standardisiert, strukturiert, synoptisch?

**Figure 1:**
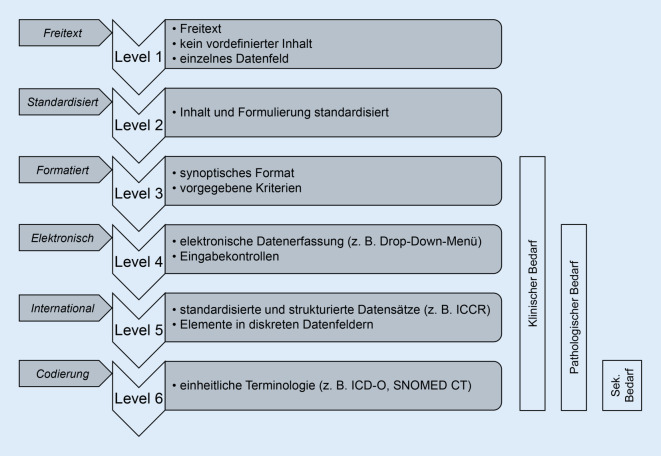

Die Verwendung der Begriffe „standardisiert“, „strukturiert“ und „synoptisch“ ist in der Literatur leider nicht ganz einheitlich. Das College of American Pathologists, welches die längste Tradition auf diesem Gebiet hat, verwendet systematisch den Begriff „synoptisch“ und bezieht in dessen Definition auch Aspekte der Formatierung ein. Nach einer Klassifikation von Ellis und Srigley [4] definiert jedoch ein „synoptisches“ Format ein mittleres Niveau der Strukturierung (Abb. 1). Für Berichte, die zusätzlich eine zugrunde liegende Datenbankstruktur haben, verwenden sie den Begriff „standardisiertes strukturiertes Berichten“.

Angesichts seiner Verbreitung in der englischsprachigen Literatur verwenden die Autoren in diesem Artikel vorzugsweise den Begriff „synoptisch“, zumal das synoptische Format das offensichtlichste Merkmal dieser Art von Berichten ist.

## Vollständigere Berichte durch synoptisches Format

Neben einer Vereinheitlichung der Terminologie ist eine verbesserte Vollständigkeit bezogen auf prognostisch oder therapeutisch wichtige Parameter das wichtigste Ziel synoptischer Berichte. Dass dieses Ziel durch die Einführung synoptischer Berichte tatsächlich erreicht wird, ist in der Literatur gut belegt [6]. Eine Vielzahl von Studien zeigt diesen Effekt beispielsweise für maligne Melanome [5–7], kolorektale Karzinome [10] oder Prostatakarzinome [1]. Eine Metaanalyse fand eine verbesserte Vollständigkeit von Berichten in 32 von 33 analysierten Studien [12]. Interessanterweise scheint ein synoptisches Format die Vollständigkeit von Berichten unabhängig davon zu verbessern, ob es sich um im entsprechenden Teilgebiet spezialisierte Pathologen handelt oder nicht [7, 10].

Für praktische Zwecke kann ferner auch schlicht der Aspekt relevant sein, dass mittels synoptischer Berichte alle relevanten Parameter an einem Ort zusammengefasst sind. Dies ist vor allem von Bedeutung, wenn die entsprechenden Proben (z. B. das resezierte Organ und die lokoregionären Lymphknoten) mit verschiedenen Antragsformularen zu verschiedenen Zeitpunkten eingesandt werden. Bei solchen parallelen Einsendungen können die synoptischen Protokolle erfahrungsgemäß sehr hilfreich zu sein, um beim Verfassen der Berichte oder der Präsentation im Tumorboard nicht die Übersicht zu verlieren.

## Quellen für synoptische Tumorprotokolle

Aktuell existieren 2 größere Organisationen, die Protokolle für synoptische Berichte publizieren. Das CAP stellt nahezu 100 verschiedene Protokolle zur Verfügung, darunter neben Protokollen für Tumorresektionen auch solche für Biopsien bzw. Biomarker. Die Protokolle werden regelmäßig aktualisiert und sind kostenfrei auf der Internetseite der CAP [3] verfügbar. Ihre Verwendung ohne Lizenzierung unterliegt jedoch bestimmten Einschränkungen im Hinblick auf die Tiefe der Softwareintegration. Die Verwendung der Protokolle für Resektate ist für CAP-akkreditierte Institute obligatorisch. Es existieren verschiedene kommerzielle US-amerikanische Anbieter von Softwareschnittstellen zu Pathologielabor-Informationssystemen, nach Kenntnis der Autoren jedoch weder in deutscher Sprache noch kompatibel mit hierzulande verbreiteten Pathologiesystemen.

Die jüngere der beiden großen Herausgeberinnen von synoptischen Protokollen ist die International Collaboration on Cancer Reporting (ICCR) [13], eine gemeinsame Initiative verschiedener nationaler und internationaler Pathologieverbände, darunter auch die Deutsche Gesellschaft für Pathologie und die Österreichische Gesellschaft für Pathologie. Bislang sind rund 50 ICCR-Protokolle veröffentlicht, weitere sind in Vorbereitung. Grundsätzlich werden die Protokolle auf Englisch verfasst, für einzelne Protokolle sind aktuell autorisierte französische, spanische bzw. portugiesische Übersetzungen verfügbar. Die Protokolle werden als PDF-Dokumente publiziert, zusätzlich sind Word-Dokumente mit dem Inhalt der Protokolle verfügbar. ICCR gestattet eine weitgehend freie Nutzung der Protokolle für diagnostische Berichte und nicht kommerzielle Forschung. Sowohl die Datensätze der ICCR als auch der CAP integrieren die Informationen aus den wichtigen tumorbezogenen Standardquellen wie den jeweils aktuellen WHO-Klassifikationen und den UICC/AJCC-TNM-Klassifikationen. Daneben werden evidenzbasierte Ergebnisse von wissenschaftlichen Publikationen eingearbeitet, die auch in den umfassenden Referenzlisten enthalten sind. Die ICCR bezieht sich zusätzlich auch auf Guidelines nationaler Fachgesellschaften, wie z. B. des Royal College of Pathologists, des Royal College of Pathologists of Australasia und auch der CAP.

CAP und ICCR entwickeln die Protokolle jeweils in einem mehrstufigen Prozess, beruhend auf aktueller Evidenz, der Arbeit von Expertengremien und einer öffentlichen Konsultationsphase. Je nach Evidenz wird zwischen obligaten und fakultativen Datenelementen unterschieden, wobei im Detail durchaus relevante Unterschiede zwischen den jeweiligen Protokollen der beiden Herausgeberinnen bestehen können. Wie bereits erwähnt, wird eine Abstimmung mit den jeweiligen Auflagen der WHO-Klassifikation und der TNM-Klassifikation angestrebt.

## Informationstechnische Umsetzung

Im Idealfall sind die Vorlagen und Datensätze in einem elektronischen Befundsystem hinterlegt und können auch elektronisch ausgefüllt werden. In der Praxis werden jedoch Pathologiebefunde häufig noch immer als Textdokument oder Text innerhalb eines in Blöcken aufgebauten Befundsystems verfasst. Die Implementierung der vorgeschlagenen Datensätze in den Pathologiebefund kann inhaltlich und formell bereits auf einer solchen Textebene erfolgen und wäre somit in Level 3 entsprechend der in Abb. 1 dargestellten Kategorisierung anzusiedeln. Für eine inhaltlich standardisierte Befundung können institutionalisierte Berichtsvorlagen verwendet oder es kann mit Textbausteinen gearbeitet werden (Beispiel – ICCR-Datensatz – Einarbeitung in Fließtext – synoptischer Bericht). Derartige Vorlagen können dann auch bereits die Grundlagen für IT-basierte Lösungen sein.

Nicht zu unterschätzen ist allerdings der Aufwand, nicht nur für eine erstmalige Implementierung eines breiten Spektrums an synoptischen Berichtsvorlagen, sondern vor allem für deren kontinuierliche Aktualisierung. Hier dürften auch die Ressourcen auch größerer Pathologischer Institute rasch überfordern sein. Außerdem wäre der betreffende Aufwand jeweils weitgehend durch die jeweilige Pathologie zu leisten, während andere Fachdisziplinen (und auch Forschung sowie Krankenhausverwaltung) mindestens im gleichen Umfang profitieren würden.

Mittel- bis längerfristig scheint daher eine gewissen Professionalisierung der betreffenden IT-Lösungen unumgänglich, ähnlich wie aktuell bereits in den USA oder auch den Niederlanden. Dabei sind die Modelle durchaus unterschiedlich. In den USA existiert eine Reihe von kommerziellen Anbietern die lizenzierte, auf den CAP-Protokollen basierende Softwarelösungen anbieten, die wiederum über Schnittstellen zu den gängigen Pathologie-Informationssystemen verfügen. Dagegen sind in den Niederlanden sowohl die Entwicklung als auch die informationstechnische Umsetzung synoptischer Berichte in der Hand eines nicht gewinnorientierten Anbieters, der Stiftung „Pathologisch-Anatomisch Landelijk Geautomatiseerd Archief“ (PALGA) [11].

Eine gewisse zusätzliche Herausforderung für eine Implementierung synoptischer Berichte im deutschsprachigen Raum ergibt sich daraus, dass bislang keine autorisierten deutschen Übersetzungen der gängigen Protokolle existieren [2]. Während die Protokolle der CAP ausschließlich auf Englisch angeboten werden, hat ICCR begonnen, offizielle Übersetzungen zu publizieren (bislang auf Spanisch, Portugiesisch und Französisch).

## ICCR-Protokolle für gastrointestinale Tumoren

In jüngerer Vergangenheit hat ICCR eine Reihe von Protokollen zu gastrointestinalen Tumoren publiziert. Zum Zeitpunkt der Verfassung dieses Manuskripts waren dies Protokolle für Resektionen von Karzinomen des exokrinen Pankreas, des Ösophagus, des Magens, des Kolons und Rektums sowie für intrahepatische/perihiläre Cholangiokarzinome bzw. hepatozelluläre Karzinome. Weiterhin existieren spezifische Protokolle für endoskopische Resektionen von Karzinomen des Ösophagus und gastroösophagealen Übergangs, des Magens und für Polypektomien aus Kolon und Rektum vor. Die Protokolle sind über die Homepage der ICCR öffentlich zugänglich und können ohne Lizenzgebühren verwendet werden. Die sukzessive Veröffentlichung der Protokolle auf der Homepage der ICCR wird von Publikationen in internationalen Fachzeitschriften begleitet [8, 9].

Ein wesentlicher Unterschied zu den CAP-Protokollen besteht darin, dass die Protokolle der ICCR noch systematischer und detaillierter klinische Daten dokumentieren, die eine prognostische oder differenzialdiagnostische Bedeutung besitzen. Hieraus könnte sich die interessante Perspektive ergeben, die betreffenden Daten bereits bei der Einsendung der Proben in standardisierter Form zu erheben. Vor allem wenn eine elegante Einbindung in Klinikinformationssysteme vorhanden wäre, würde dies nicht nur uns Pathologen unsere Arbeit erleichtern, sondern einen echten Mehrwert für die Qualität der Patientenversorgung bedeuten.

**Figure 2:**
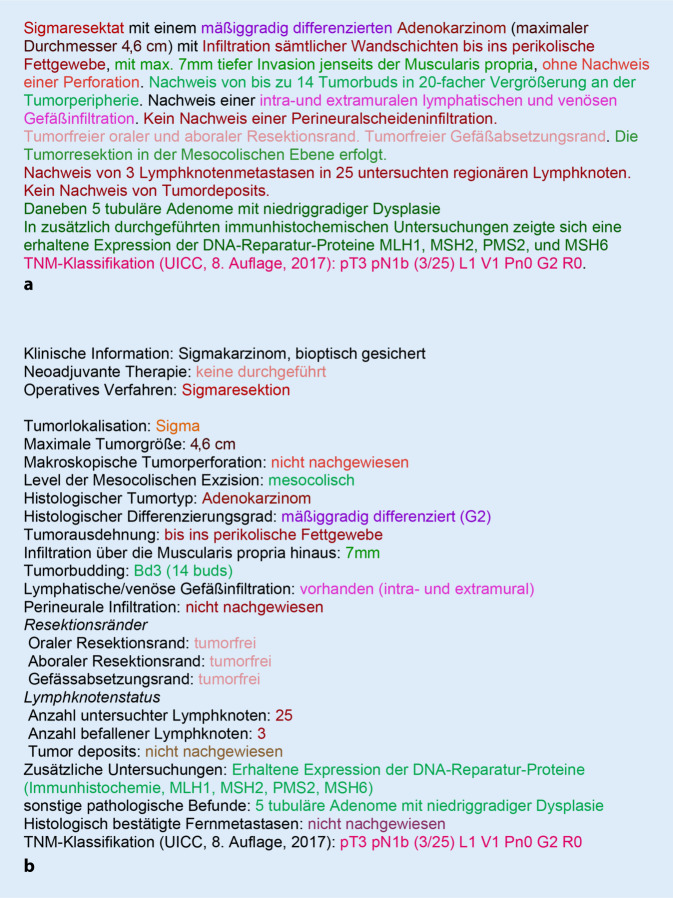

Die ICCR-Protokolle unterscheiden obligate Parameter („core items“) von fakultativen („non-core items“). In Tab. 1 wird dies am Beispiel des Protokolls für kolorektale Karzinome dargestellt; Abb. 2 illustriert anhand des Beispiels eines Sigmakarzinoms eine mögliche konkrete Umsetzung des betreffenden ICCR-Protokolls im Vergleich zu einem inhaltlich übereinstimmenden narrativen Bericht.

**Table 1:** 

Elemente des synoptischen Formats für das kolorektale Karzinom (ICCR-Dataset)
*Obligate („core“) Elemente*
Neoadjuvante Therapie
Operatives Verfahren
Tumorlokalisation
Maximale Tumorgröße
Makroskopische Tumorperforation
Bezug zur anterioren peritonealen Umschlagfalte^a^
Ebene der mesorektalen Exzision^a^
Histologischer Tumortyp
Histologischer Differenzierungsgrad
Tumorausdehnung
Lymphatische/venöse Gefäßinfiltration
Perineurale Infiltration
Lymphknotenstatus
Tumor deposits
Ansprechen auf neoadjuvante Therapie
Resektionsstatus
Histologisch bestätigte Fernmetastasen
TNM-Klassifikation
*Fakultative („non-Core“) Elemente*
Klinische Informationen
Ebene der Sphinkterexzision^a^
Ebene der mesokolischen Exzision
Tumorausbreitung über die Muscularis propria hinaus
Tumor Budding
Sonstige pathologische Befunde
*Ergänzende immunhistochemische Informationen (nur Coreelement für neuroendokrine Neoplasien)*

*ICCR* International Collaboration for Cancer Reporting

^a^nur relevant beim rektalen Karzinom

## Ausblick

Bereits die ersten Schritte in Richtung stärker strukturierter und standardisierter Berichte (bis Stufe 3 nach Ellis und Srigley [4]) bieten einen Nutzen für den jeweiligen Patienten und das für seine Behandlung verantwortliche interdisziplinäre Team. Besonders deutlich wird dies im Rahmen des interdisziplinären Tumorboards, wo die Vollständigkeit der Berichte und deren eindeutige Terminologie entscheidend zu einer optimalen Entscheidungsfindung beitragen können. Nebenbei bemerkt ist es umso erfreulicher, dass das Konzept der synoptischen Berichte von der Pathologie auch auf andere Disziplinen überzugreifen beginnt [6].

Ein erheblich über den einzelnen Patienten hinausreichendes Potenzial ergibt sich hingegen vor allem durch die Stufen 4–6 nach Ellis and Srigley [4], da sich hieraus wesentliche Impulse für die Qualitätssicherung, sowie über eine Integration mit epidemiologischen Krebsregistern und Biobanken auch für verschiedenste wissenschaftliche Fragestellungen ergeben.

## Fazit für die Praxis

- Synoptische Berichte tragen zur Standardisierung und verbesserten Vollständigkeit von Pathologiebefunden bei.
- International Collaboration for Cancer Reporting (ICCR) und College of American Pathologists (CAP) stellen eine Vielzahl aktueller und evidenzbasierter Protokolle zur Verfügung.
- Die praktische Umsetzung im deutschsprachigen Raum ist zurzeit noch anspruchsvoll, da autorisierte deutsche Übersetzungen bislang fehlen.
- Auch wer synoptische Berichte (noch) nicht verwendet, findet in den Protokollen eine Fülle an aktuellen und relevanten Informationen zu den betreffenden Tumortypen.
