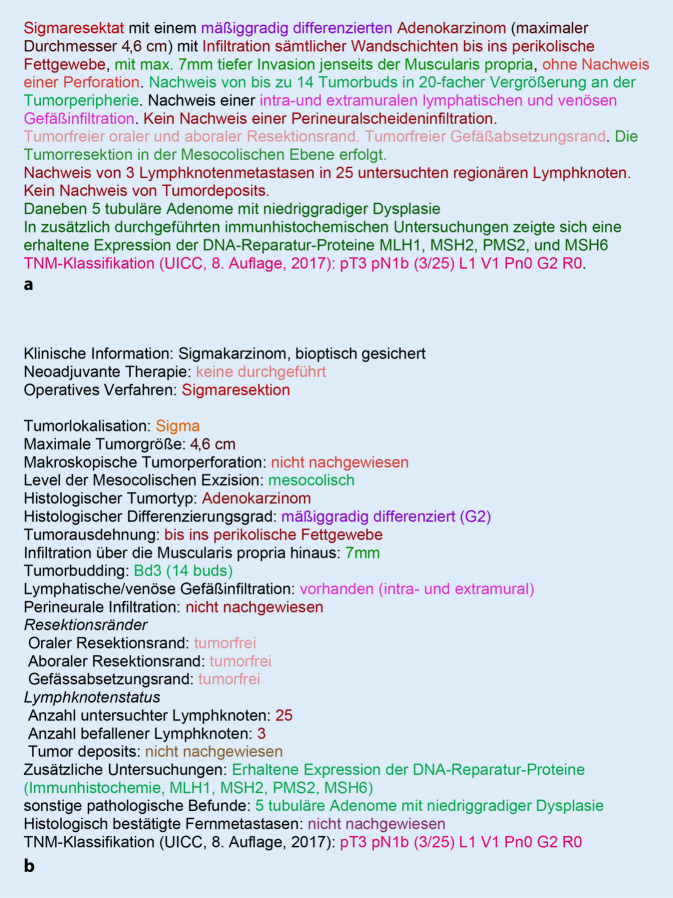# Erratum zu: Standardisierte strukturierte Befundberichte gastrointestinaler Tumoren

**DOI:** 10.1007/s00292-021-01020-w

**Published:** 2021-11-25

**Authors:** Ekkehard Hewer, Anna Rump, Rupert Langer

**Affiliations:** 1 Institut universitaire de pathologie, Centre hospitalier universitaire vaudois (CHUV) et Université de Lausanne, Rue du Bugnon 25, 1011 Lausanne, Schweiz; 2 Institut für Pathologie und Molekularpathologie, Kepler Universitätsklinikum und Johannes-Kepler-Universität, Linz, Österreich

**Erratum zu:**

**Pathologe 2021**

10.1007/s00292-021-00986-x

In Abb. 2 in diesem Artikel wurde die Größenangabe zur maximalen Tumorgröße falsch wiedergegeben. Die Abbildung hätte wie folgt aussehen sollen. Der Originalbeitrag wurde korrigiert.

**Figure 1:**